# Phytochemical analysis, antioxidant, and antimicrobial activities of Jordanian Pomegranate peels

**DOI:** 10.1371/journal.pone.0295129

**Published:** 2023-11-30

**Authors:** Nuha Sweidan, Walid Abu Rayyan, Iman Mahmoud, Leen Ali

**Affiliations:** 1 Faculty of Arts and Sciences, Department of Chemistry, University of Petra, Amman, Jordan; 2 Faculty of Science, Department of Medical Laboratory Analysis, Al-Balqa Applied University, Al-salt, Jordan; 3 Faculty of Pharmacy, Department of Nutrition, University of Petra, Amman, Jordan; Universidad Autónoma de Coahuila, MEXICO

## Abstract

Pomegranate (*Punica granatum*) peels have shown numerous health benefits such as antioxidant, anti-inflammatory, and antimicrobial activities. These health activities are owed to the unique phytochemical components present in pomegranate peels. Variations in the pomegranate cultivar, geographical region, and extraction methods significantly affect the phytochemical composition and concentrations of pomegranate fruits and their peels, hence their health outcomes. Therefore, this study aimed to examine the phytochemical contents of pomegranate peels of Jordanian origin and their antioxidant and antimicrobial activities. Among the 6 extracts of pomegranate peels tested, the ethanol extract exhibited the highest total phenolic content (TPC = 297.70 ± 1.73 mg GAE/g DW), highest total flavonoids content (TFC = 116.08 ± 3.46 mg RE/g DW), highest hydrolyzable tannins (HT) contents (688.50 ± 3.54 mg TE/g DW). Whereas the highest condensed tannins (CT) content was found in both the ethanol (13.87 ± 0.58 mg CE/g DW) and methanol (13.84 ± 0.55 mg CE/g DW) extracts. For the antioxidant activities, the water extract of pomegranate peels displayed the highest inhibitory effect on DPPH radicals (9.43 ± 0.06 μmole TE/g DW), while for the ABTS^+^ assay the methanol and ethanol extracts exhibited the highest activities of 11.09 ± 0.02 and 11.09 ± 0.06 μmole TE/g DW, respectively. For the FRAP assay, the aqueous methanol extract exhibited the highest reducing activity (1.60 ± 0.09 mmole Fe (II)/g DW). As for the antimicrobial activities of various extracts of pomegranate peels, the highest antimicrobial activity against *Micrococcus luteus* was achieved by the ethanol extract (MIC = 6.25 mg/mL), whereas the lowest antimicrobial activity was observed against *Candida krusei* using the methanol extract (MIC = 100 mg/mL). These results indicate that pomegranate peels of Jordanian origin are rich in phytochemical content and exhibited strong antioxidant and antimicrobial activities making these agroindustrial by-products potential candidates for various medical applications and possible safe sources for important bioactive components.

## Introduction

The pomegranate (*Punica granatum*) is a fruit tree that belongs to the Lythraceae family. Originating from the Mediterranean region, pomegranate has been used extensively in traditional medicine as a natural antiviral, antifungal, and antibacterial fruit. In ancient times, pomegranate juice has been used as a natural source for treating diarrhea and harmful internal parasites [1]. The rise in the consumption of pomegranate juice has led to the high production of its peels which makes up to 60% of pomegranate fruit weight [2]. This abundant by-product has been reported to reduce the risk of many chronic human diseases such as breast [3], colon [3–5], and lung cancer [6]. The anti-inflammatory potential of the peels and peel extracts have also been reported [7–9]. Several *in vivo* and *in vitro* studies have also demonstrated the vital role of pomegranate peels in cardiovascular diseases [10, 11]. Recently, pomegranate peel extracts have been used as potential natural food preservatives due to its antimicrobial and antioxidant capacities [12].

The diversity in the medicinal functions found in pomegranate peels has been attributed to their reported bioactive components. A review by Chen et al. [13] reported the isolation of almost 49 bioactive compounds from pomegranate peels with the majority being phenolic acids, flavonoids, and tannins. The unique chemical structures of these bioactive compounds have been reported to possess numerous beneficial biological activities such as antioxidants, antimicrobial, anticancer, anti-inflammatory activities that attracted the attention of chemists, biologists, nutritionists, and health care scientists worldwide [3, 14, 15].

Despite the well-documented chemical composition of pomegranate peels, particularly the bioactive phytochemical compounds, many factors have been shown to significantly affect the quality and quantity of these compounds, hence their related health activities. Cultivar variations, geographical region, farming conditions, and experimental conditions are major factors affecting the bioactive composition of pomegranate peels [13, 15]. Bassiri-Jahromi and Doostkam [16] reported that the contents of both phenolic and flavonoids differed significantly in various pomegranate peel cultivars found in different world regions. Other studies showed a wide variations in the physico-chemical properties of pomegranate peels extracted by various methods and different solvents [17, 18]. A recent study by Altarawneh et al. [18] showed that different solvents yielded different total phenolic content (TPC) and total flavonoid content (TFC) and produced various antioxidant activities. They also reported that fractions of the extracts gave different bioactive components’ quantities and qualities than that found in their crude extracts. The authors concluded the need for further investigations on the Jordanian pomegranate peels and their bioactive components as well as their various biological activities.

Hence, the present study aimed to examine the qualitative and quantitative (total phenols, flavonoids, and hydrolyzable and condensate tannins) composition of Jordanian pomegranate peels extracted by 6 different solvents. Moreover, the antioxidant activities (DPPH, ABTS^+^, and FRAP) and antimicrobial capacities (against Gram-positive and Gram-negative bacteria and fungi) of these extracts were investigated.

## Materials and methods

### Plant materials

Pomegranate fruits were harvested in October 2022 from pomegranate trees located in Jerash (60 km north of Amman). The pomegranate peels were manually removed from the fruits and were washed twice with double distilled water (ddH_2_O) and dried in the shade until complete dryness was achieved. Dried peels were then crushed into a coarse powder with a grain grinder (the size of the powder was 10 mesh or less). The resultant powdered samples were kept in airtight containers, in dark until further analysis.

### Extraction

Extract yields of the same plant may vary widely upon the usage of different extraction mechanisms. Extraction factors such as temperature, the polarity of solvent used for extraction, extraction time, and particle size of samples significantly influence the quality and quantity of the sample’s bioactive components [19]. Temperatures of 20–50°C, shorter time intervals, usage of solvents of various polarities, and smaller sample particle size have been considered as sufficient and efficient for the extraction of the bioactive components and their antioxidant activities from plant sources [20]. In brief, pomegranate peel extracts were prepared by soaking 10g of peels’ powder in 100 mL solvents of various polarity. The 6 extraction solvents used in this study were water, methanol, aqueous methanol (70% methanol/ 30% water), ethanol, ethyl acetate, and butanol. The powder-solvent solutions were stirred at 35°C for 4 hours using a shaking incubator at a speed of 100 rpm (Stuart TM, SBS40, China). Extracts were then filtered with Whatman no. 1 filter paper. The filtrates were evaporated under a vacuum till dryness using a rotary evaporator (Stuart Diagonal Condenser–RE400 & RE400P, China). The water extract was evaporated under room temperature until a constant weight was achieved (complete dryness was achieved after 4 days). The resultant dried extracts in the form of resin were kept at 4°C in amber vials.

### Materials

Gallic acid, tannic acid, and Folin-Cioalteu’s phenol reagent were purchased from Sigma Chemical Co.(St. Louis, MO, USA). Trolox and Rutin were purchased from (Aldrich, Milwaukee, WI). All chemicals used in antioxidant activities were HPLC grades (Sigma Chemical Co. (Poole, Dorse). Spectrophotometric measurements were performed on a Rayleigh ultraviolet (UV)- 2601 spectrometer (Bio-Equip, Beijing, China).

### Phytochemical contents and antioxidant activities

#### Qualitative phytochemical screening

Phytochemical screening of key families of pomegranate peel extracts was performed according to the methods reported by Trease and Evans [21] and Sakar and Tanker [22]. In brief, polyphenols and tannins were tested using Braymer’s test (ferric chloride in HCl), while the presence of flavonoids was analyzed using the Shinoda’s test (metallic magnesium and hydrochloric acid). Saponins were tested for their ability for foam formation using the Frothing test. The presence of quinones in tested extracts was evaluated by the ferric chloride test, whereas steroids were screened using Liebermann-Burchard’s test. Anthocyanins and coumarins contents were detected using a sodium hydroxide solution. Finally, triterpenes and alkaloids were screened by applying acetic anhydride/ sulfuric acid and Dragendroff’s tests, respectively [23].

#### Determination of total phenolic content

The TPC of pomegranate peel extracts was determined using the Folin-Ciocalteu reagent according to the method reported by Li et al. [24]. The results were expressed as milligram gallic acid equivalents per gram sample dry weight (mg GAE/g DW). In brief, 1.7 mL of 10% Folin-Ciocalteu reagent diluted with ddH_2_O was mixed with 0.3 mL of each tested extract and incubated at room temperature for 10 min. The intensity of the resultant blue color was measured at an absorbance of 760 nm using a Rayleigh 2601-UV Spectrophotometer. A triplicate for each sample was made.

#### Determination of total flavonoids content

The TFC was calculated using the Rutin calibration curve (0.01–0.1 mg/mL) and was reported in mg of Rutin equivalent per g of extract (mg RU/g extract). The procedure followed that reported by Djeridane et al. [25]. In brief, 1 mL of peel extracts was mixed with 0.5 mL of 10% methanolic aluminum chloride, followed by the addition of 0.5 mL of 1 M HCl and incubated for 30 min at room temperature. The absorbance was measured at 425 nm using a Rayleigh 2601-UV Spectrophotometer. The result was expressed as mg Rutin equivalents per g dry weight (mg RE/g DW).

#### Determination of total condensed tannins content

The CT contents were determined by the vanillin assay with slight modifications [26]. One mL of the extracts was mixed with 2.5 mL of 4% vanillin in methanol and then mixed with 2.5 mL of 25% H_2_SO_4_ in methanol to undergo a vanillin reaction. The mixture was allowed to stand for 15 min in the dark at room temperature. The absorbance of the mixture was measured at 500 nm. Different concentrations of catechin solutions (10–100 mg/L) were used for the standard curve. The final results were expressed as mg catechin equivalent per g of extract dry weight (mg CE/g DW).

#### Determination of total hydrolyzable tannins content

The HT contents were determined by the method of Çam and Hişil [27]. One mL of each extract was added to 5 mL of 2.5% KIO_3_ and the mixture was vortexed for 10 seconds (s). The absorbance of the developed red color was measured at 550 nm. Serial dilutions of tannic acid solution (50–1500 mg/L) were used for the calibration of the standard curve. The final results were expressed as mg tannic acid equivalent per g of extract dry weight (mg TAE/g DW).

#### Determination of DPPH radical scavenging activity

The radical-scavenging activity of different extracts of DPPH radical was determined according to the method reported by ElFalleh et al. [14]. DPPH solution (100 μM) was mixed with 100 μL sample solutions at different concentrations (0.025–1.0 mg/mL) of the different extracts. All solutions obtained were shaken vigorously and then incubated in the dark for 1 hour at 25°C. Absorbances were measured at 517 nm. The control solution was prepared from 100 μM DPPH dissolved in methanol. Trolox (25–200 mg/mL) was used for calibrations. The radical scavenging capacity using the free DPPH radicals was evaluated by measuring the decrease of the absorbance at 517 nm. Results were expressed as micromolar Trolox equivalent per g extract dry weight (μmole TE/g DW).

#### Determination of ABTS^+^ radical scavenging activity

The ABTS^+^ assay was performed based on the procedure established by Re et al. [28]. To 10μL Trolox (25–200 mg/mL) standard or extract, 2 mL ABTS reagent (7 mM +2.45 mM persulphate solution with a ratio 2:1) was added and incubated for 10 min in dark at room temperature. Then extracts were read at 734 nm using a 96-well reader (thermoscientific Multiskan SKY, USA). Results were expressed as micromolar Trolox equivalent per g extract dry weight (μmole TE/g DW).

#### Determination of ferric reducing antioxidant power (FRAP)

The ferric-reducing antioxidant power (FRAP) assay was performed following the method by Benzie and Strain [29] and was adjusted for 96-well plates. Briefly, fresh FRAP reagent was prepared from 10 mL sodium acetate buffer (300 mM in glacial acid), 1 mL 2,4,6-tri(2-pyridyl)-s-triazine 99% (TPTZ, 10 mM in 40 mM HCl) and 1 mL iron (III) chloride hexahydrate solution (20 mM). Subsequently, 10 μL of the various extracts (1 mg/mL) were mixed with 200 μL FRAP reagent and the absorbance was read at 4 min after the addition of the FRAP reagent, using a microplate reader (593 nm). Ferric sulfate heptahydrate (0–1000 μg/mL) was used as the standard curve. FRAP value was expressed as millimoles ferrous ion per g dry weight extract (mmole Fe (II)/g DW).

### Antimicrobial activity

#### Organisms and cultural conditions

Seven bacterial strains; three Gram-negative bacteria; *Salmonella typhi* (ATCC 1331), *Pseudomonas aeruginosa* (ATCC 9027), and *Klebsiella pneumoniae* (ATCC 700603), and two Gram-positive bacteria; *Streptococcus pyogenes* (ATCC 19615) and *Micrococcus luteus* (ATCC 10240) and two fungal strains; *Candida albicans* (ATCC 10231) and *Candida krusei* (ATCC 6258) were procured from the American Type Culture Collection (ATCC, USA). Nutrient broth (for bacterial strains) and potato dextrose broth (for fungal strains) were purchased from Bio Lab (Hungary). Ciprofloxacin (antibacterial agent) and fluconazole (antifungal agent) were purchased from GenHunter (Germany). The seven opportunistic and pathogenic microorganisms were utilized in the antimicrobial screening assay for the six pomegranate peel extracts. The bacterial strains were preserved using glycerin stocks and stored for the long term at -80°C. Bacterial strains were cultivated and propagated using nutrient broth at 37°C [30], whereas fungal strains were cultured in potato dextrose broth at 30°C [31].

#### Agar diffusion assay

To examine the antimicrobial activity for the six tested extracts, an overnight broth culture of the Gram-positive bacteria *Streptococcus pyogenes* and *Micrococcus luteus*, and Gram-negative bacteria strains *Salmonella typhi*, *Pseudomonas aeruginosa*, and *Klebsiella pneumoniae* were inoculated individually and diluted to 4 x 10^6^ cfu/mL in 100 mL of nutrient broth at 37°C for 16–18 hours. On the other hand, two fungal strains *Candida albicans* and *Candida krusei* were cultured in potato dextrose broth at 30°C for 48 hours.

For agar diffusion assay [30], three cm of Muller Hinton agar was poured into 9 cm plastic Petri dishes to examine the antibacterial activities of the extracts, whereas potato dextrose agar was used for the antifungal examination. The poured medium in Petri dishes were left to solidify for 30 min at room temperature. Aliquots of 200 μL of overnight bacterial cultures were dispensed on agar plates. Then, 1.5 mm wells were punched in the solidified agar and filled individually with 100 μL of the water diluted extracted solutions. The extracts were diluted by mixing 200 μL of the extract with 1.8 mL of water. Plates were incubated for 16–18 hours at 37°C for bacterial strains and at 30°C for 48 hours for fungal strains. Ciprofloxacin was used as a reference drug for the antibacterial examination, whereas fluconazole was the positive standard for the antifungal examination. After the incubation period, inhibition zones were measured and recorded for further analysis.

#### Broth microdilution assay

For the veracity of the antimicrobial screening of the six pomegranate peel extracts (water, methanol, aqueous methanol (70% methanol/ 30% water), ethanol, ethyl acetate, and butanol), broth assays in 96-well microplates were adopted [32–34]. A serial of two-fold dilution for the six extracts as well as ciprofloxacin and fluconazole were added to the 96-well microplate rows. The final concentrations of the extracts ranged between 1.5 mg/mL to 100 mg/mL in each well. The optimal concentration for each well was reached by conducting a serial dilution of 100 μL of the extract mixed with 100 μL of nutrient broth. Each row contained one single type of bacteria of the test set (*Streptococcus pyogenes*, *Micrococcus luteus*, *Salmonella typhi*, *Pseudomonas aeruginosa*, and *Klebsiella pneumoniae*) as well as ciprofloxacin to the final row as a positive control. The plates were then covered and incubated at 37°C for 16–18 hours. On the other hand, fungal strains (*Candida albicans* and *Candida krusei*) were pipetted with potato dextrose broth to each well, and fluconazole was added to the final row as a positive control, then plates were covered and incubated at 30°C for 48 hours. Afterward, the minimum inhibitory concentration (MIC) values were determined by reading the optical density at 540 nm using a 96-well reader (Thermoscientific Multiskan SKY, USA).

### Statistical analysis

Statistical analyses were performed using SPSS version 20 (IBM Corp. Armonk, NY, USA). All analyses were carried out in at least three replicates. Data were expressed as mean ± SD (Standard deviation). Statistical significance was determined by two-way ANOVA followed by Bonferroni’s multiple comparison test to determine the statistical significance among the tested groups and were considered significant at *P* < 0.05, unless otherwise stated.

## Results

### Qualitative phytochemical screening

Different phytochemical classes were detected in all extracts of pomegranate peels (Table 1). As demonstrated in Table 1, the ethanol extract of pomegranate peels showed positive detection to all phytochemical classes tested (phenols, flavonoids, anthocyanins, coumarins, quinones, tannins, saponins, steroids, triterpenoid, and alkaloids). On the other hand, the ethyl acetate extract showed negative results for phenols, flavonoids, anthocyanins, steroids, and triterpenoids. The butanol, methanol, aqueous methanol (70% methanol/ 30% water), and water extracts exhibited positive results for the various phytochemical classes detected with the exception for steroids and triterpenoids.

**Table 1:** Qualitative phytochemical screening of various extracts of pomegranate peels.

Test/ Extract	EtoAc	Butanol	Methanol	Aq methanol (70%methanol/ 30%water)	Water	Ethanol
**Phenols**	-ve	+ve	+ve	+ve	+ve	+ve
**Flavonoids**	-ve	+ve	+ve	+ve	+ve	+ve
**Anthocyanins**	-ve	+ve	+ve	+ve	+ve	+ve
**Coumarins**	+ve	+ve	+ve	+ve	+ve	+ve
**Quinones**	+ve	+ve	+ve	+ve	+ve	+ve
**Tannins**	+ve	+ve	+ve	+ve	+ve	+ve
**Saponins**	+ve	+ve	+ve	+ve	+ve	+ve
**Steroids**	-ve	-ve	-ve	-ve	-ve	+ve
**Triterpenoid**	-ve	-ve	-ve	-ve	-ve	+ve
**Alkaloids**	+ve	+ve	+ve	+ve	+ve	+ve

EtOAc = Ethyl acetate; Aq methanol = Aqueous methanol; +ve = Positive detection; -ve = Negative detection.

### Quantitative contents of phenolic, flavonoids, hydrolyzable and condensed tannins

Table 2 displays the TPC, TFC, HT, and CT of the various extracts of pomegranate peels. Regarding the TPC, the ethanol extract exhibited the highest content (297.70 ± 1.73 mg GAE/g DW), while the lowest TPC content was found in the ethyl acetate extract (121.38 ± 3.51 mg GAE/g DW). For TFC, again the ethanol extract showed the highest content (116.08 ± 3.46 mg RE/g DW), while the lowest content was detected in the ethyl acetate extract (58.81 ± 0.82 mg RE/g DW). The highest content of HT was found in the ethanol extract (688.50 ± 3.54 mg TE/g DW), followed by the butanol extract (540.17 ± 8.84 mg TE/g DW), while the lowest HT content was detected in the water extract (160.17 ± 2.36 mg TAE/g DW). On the other hand, the highest CT contents were recorded in both ethanol (13.87 ± 0.58 mg CE/g DW) and methanol (13.84 ± 0.55 mg CE/g DW) extracts, however, water extract exhibited the lowest CT content (2.69 ± 0.18 mg CE/g DW; Table 2).

**Table 2:** Total phenolic content, total flavonoid content, hydrolyzable and condensed tannins of various extracts of pomegranate peels.

Extracts	TPC (mg GAE/g DW)	TFC (mg RE/g DW)	HT (mg TE/g DW)	CT (mg CE/g DW)
**Ethanol**	297.70±1.73^a^	116.08 ±3.46^a^	688.50±3.54^a^	13.87±0.58^a^
**Methanol**	237.20 ±0.96 ^bc^	97.36±1.78^b^	481.83±7.07^b^	13.84±0.55^a^
**Aq Methanol (70%methanol/ 30%water)**	257.36 ±0.35^ab^	94.37 ±1.20^bc^	473.5±7.07 ^b^	8.73±0.58^bc^
**Water**	208.42 ±3.55^c^	85.27 ±7.16^cd^	160.17±2.36^c^	2.69±0.18^d^
**Butanol**	224.24 ±2.95^c^	79.86 ±2.69^d^	540.17±8.84^ab^	6.91±0.27^c^
**EtoAc**	121.38 ±3.51^d^	58.81 ±0.82^e^	458.5±7.07^b^	6.12±0.40^c^

Values are expressed as mean ± SD (n = 3). Superscript letters with different letters in the same column indicate a significant difference (*P* < 0.05). TPC = Total phenolic content; TFC = Total flavonoid content; HT = Hydrolyzed tannins; CT = Condensed tannins; GAE/ g DW = Gallic acid equivalents/ gram dry weight of sample; RE/ g DW = Rutin equivalents/ gram dry weight of sample; TE/g DW = Trolox equivalents/ gram dry weight of sample; CE/ g DW = Catechin equivalents/ gram dry weight of sample; Aq methanol = Aqueous methanol; EtOAc = Ethyl acetate.

### Antioxidant activities of the extracts of pomegranate peels

The antioxidant activities of pomegranate peel extracts were evaluated in terms of DPPH and ABTS^+^ radical scavenging assays, as well as the FRAP assay (Table 3). For DPPH radical scavenging activity, the water extract of pomegranate peels displayed the highest scavenging effect on DPPH radicals (9.43 ± 0.06 μmole TE/g DW), followed by the methanol (8.51 ± 0.51 μmole TE/g DW) and ethanol (7.27 ± 0.85 μmole TE/g DW) extracts. Meanwhile, methanol, ethanol, butanol, and aqueous methanol extracts exhibited the highest ABTS^+^ radical scavenging activities of 11.09 ± 0.02, 11.09 ± 0.06, 10.93 ± 0.06, and 10.69 ± 0.06 μmole TE/g DW, respectively (Table 3). As for the FRAP assay, the aqueous methanol (70% methanol/ 30% water) extract exhibited the highest reducing activity (1.60 ± 0.09 mmole Fe (II)/g DW) followed by the water extract (1.44 ± 0.06 mmole Fe (II)/g DW; Table 3). Ethyl acetate extract, on the other hand, did not show a detectable radical scavenging activity against DPPH and produced the lowest radical scavenging activity against ABTS^+^ and ferric-reducing capacity (Table 3).

**Table 3:** Radical scavenging and reducing properties of various extracts of pomegranate peels.

Extract	DPPH (μmole TE/g DW)	ABTS^+^ (μmole TE/g DW)	FRAP (mmole Fe II/ g DW)
**Ethanol**	7.27 ± 0.85^a^	11.09 ± 0.06^a^	1.11 ± 0.05^a^
**Methanol**	8.51 ± 0.51^b^	11.09 ± 0.02^a^	0.93 ± 0.03^b^
**AqMethanol (70%methanol/ 30%water)**	6.79 ± 0.28^c^	10.69 ± 0.06^b^	1.60 ± 0.09^c^
**Water**	9.43 ± 0.06^d^	7.21 ± 0.06^c^	1.44 ± 0.06^d^
**Butanol**	4.91 ± 0.51^e^	10.93 ± 0.06^b^	0.92 ± 0.04^b^
**EtoAc**	ND	1.42 ± 0.94^d^	0.86 ± 0.02^e^

Values are expressed as mean ± SD (n = 4). Superscript letters with different letters in the same column indicate a significant difference (*P* < 0.05). DPPH = 2,2 diphenyl-1-picrylhydrazyl radical scavenging assay; μmole TE/g DW = Micromolar Trolox equivalents/ gram dry weight of sample; ABTS^+^ = 2,2’-azino-bis (3-ethylbenzothiazoline-6-sulfonic acid) radical scavenging assay; FRAP = Ferric reducing antioxidant power assay; mmole Fe (II)/g DW = Millimolar ferrous ions (II)/ gram dry weight of sample; Aq methanol = Aqueous methanol; EtOAc = Ethyl acetate, NA = Not detected.

### Correlation between phenolic content, flavonoid content and antioxidant activities

The Pearson’s correlation coefficients of TPC, TFC, HT, CT and the antioxidant activities (DPPH, ABTS^+^, and FRAP) are shown in Table 4. A positive and negative correlation was observed between TPC, TFC, HT, CT and the antioxidant activities of DPPH, ABTS^+^, and FRAP. The correlation ranged from weak (*r* = 0.22) to very strong correlation (*r* = 0.96). However, a significant positive correlation was detected only between TPC and TFC (*r* = 0.96; *P* < 0.01) and between TPC and ABTS^+^ (*r* = 0.91; *P* < 0.05; Table 4).

**Table 4:** Pearson’s correlation coefficients of TPC, TFC, HT, CT and antioxidant activities (DPPH, ABTS^+^, and FRAP).

Test	TPC	TFC	HT	CT	DPPH	ABTS^+^	FRAP
**TPC**	1.00						
**TFC**	0.96**	1.00					
**HT**	0.44	0.42	1.00				
**CT**	0.61	0.70	0.77	1.00			
**DPPH**	0.71	0.73	-0.26	0.22	1.00		
**ABTS** ^**+**^	0.91*	0.81	0.38	0.56	0.71	1.00	
**FRAP**	0.38	0.31	-0.42	-0.28	0.53	0.24	1.00

* and ** Correlation is significant at *P* < 0.05 and *P* < 0.01 level, respectively. TPC = Total phenolic content; TFC = Total flavonoid content; HT = Hydrolyzed tannins; CT = Condensed tannins; DPPH = 2, 2-diphenyl-1-picrylhydrazyl; ABTS^+^ = 2,2-azinobis-(3- ethylbenzothiazoline-6-sulfonate); FRAP = Ferric reducing antioxidant power.

### Antimicrobial activity of pomegranate peel extracts

#### Agar diffusion assay

**Fig 1:**
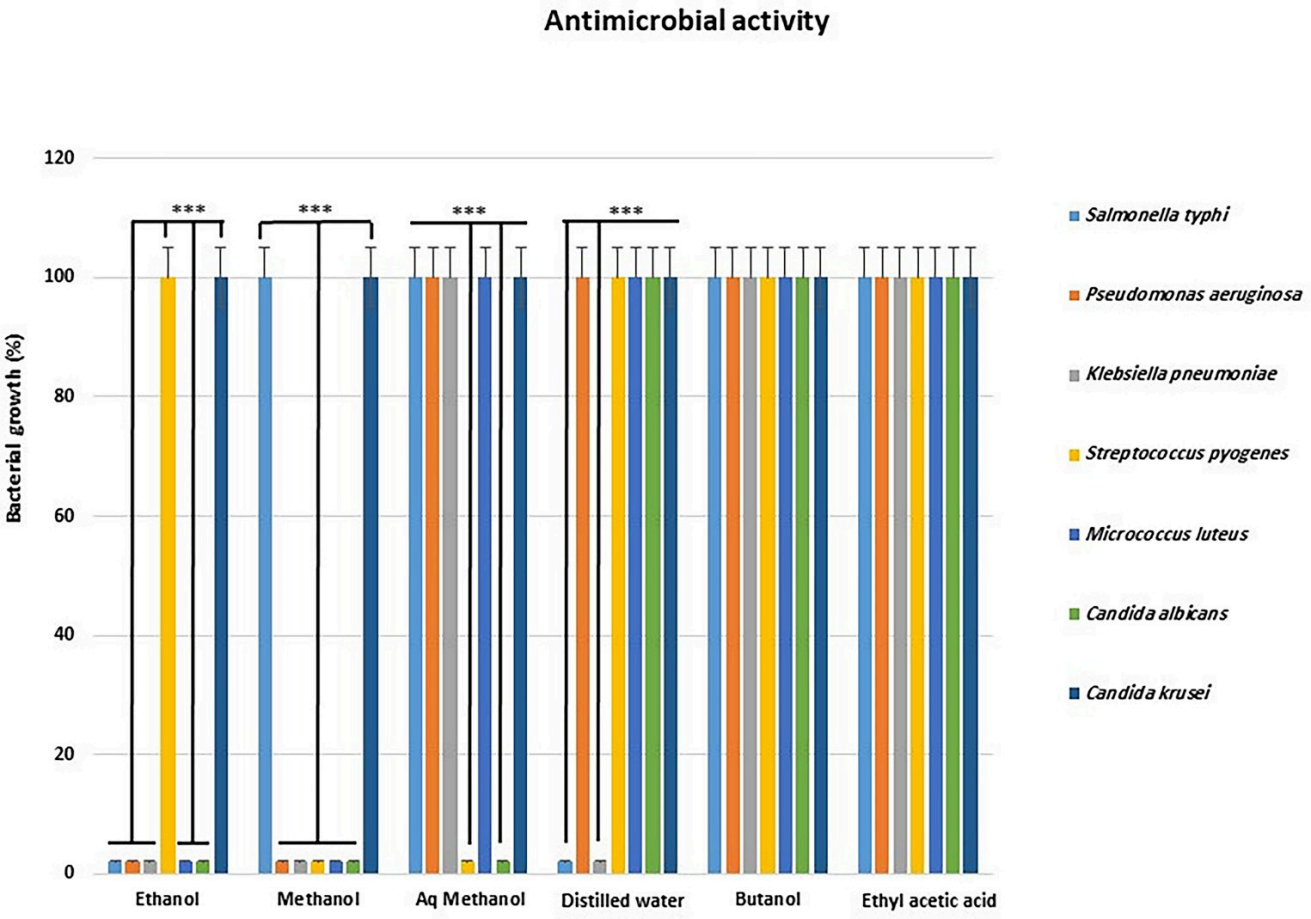
Antimicrobial activities of pomegranate peel extracts as a percentage of microbial growth inhibition against five bacterial and two fungal strains tested. ***P < 0.*001*, Aq methanol = Aqueous methanol.

The antimicrobial activities of the six pomegranate peel extracts were evaluated according to the intensity of the bacterial growth in the wells punched in the solidified agar after the 16–18 hours incubation period for bacterial strains and 48 hours for fungal strains. The ethanol and methanol extracts showed the highest inhibition activities among the six extracts tested against the bacterial and fungal strains as both inhibited five out of seven microbial strains tested (Fig 1). Both extracts have individually inhibited the bacterial strains; *Pseudomonas aeruginosa*, *Klebsiella pneumoniae*, and *Micrococcus luteus* and the fungal strain; *Candida albicans*, whereas they didn’t show any inhibition activity against the fungal strain *Candida krusei*. Solely, the ethanol extract showed inhibition activity against *Streptococcus pyogenes* and the methanol extract showed inhibition activity against *Salmonella typhi*. Whereas extract of aqueous methanol (70% methanol/ 30% water) showed inhibitory activity against *Streptococcus pyogenes* and *Candida albicans*. Meanwhile, the water extract showed antibacterial activity against only two strains; *Salmonella typhi* and *Klebsiella pneumoniae*. Butanol and ethyl acetate extracts, on the other hand, did not show any antimicrobial activities against the bacterial or fungal strains tested at the different concentrations used (1.5 mg/mL to 100 mg/mL; Fig 1).

#### Broth microdilution assay

**Fig 2:**
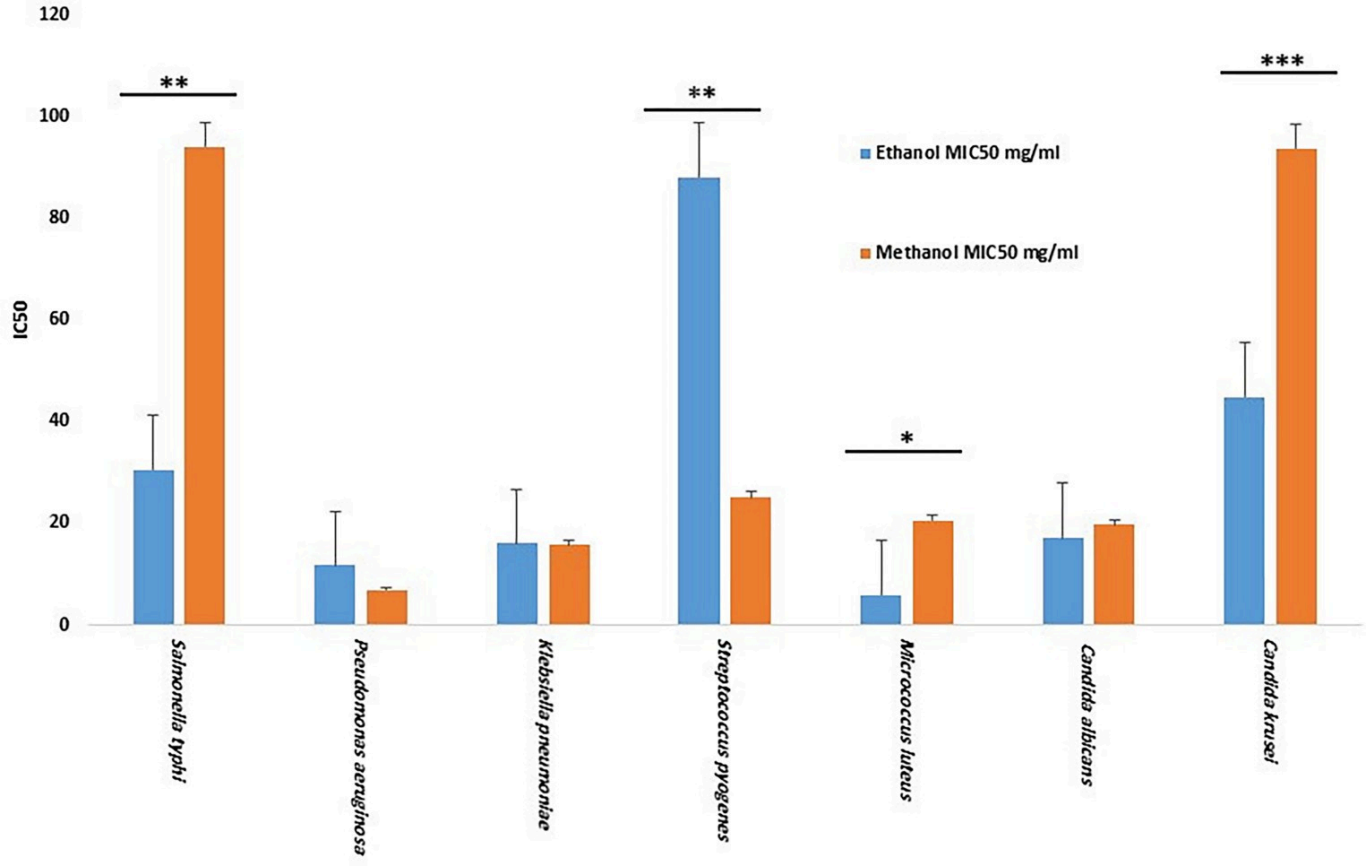
Minimum inhibition activities of the ethanolic and methanolic extracts against a set of microbial strains. *P < 0.*05*, **P < 0.*01*, ***P < 0.*001*, IC50 = The half maximal inhibitory concentration, MIC50 = The minimum inhibitory concentration 50%.

Using the broth microdilution assay, the results were homogeneous with the agar diffusion results. Hence, only the ethanol and methanol extracts were further tested. The highest antimicrobial activity was accounted to the ethanol extract as it exhibited the highest antibacterial activity against *Micrococcus luteus* producing a MIC50 of 6.25 mg/mL, followed by *Pseudomonas aeruginosa*, *Klebsiella pneumoniae*, and *Salmonella typhi*. Additionally, antifungal activity has been demonstrated by the ethanol extract against *Candida albicans* (MIC50 = 17.25 mg/mL), meanwhile, there was no antifungal inhibition detected against *Candida krusei*. On the other hand, the highest antimicrobial activity for the methanol extract was seen against *Pseudomonas aeruginosa* (MIC50 = 7.5 mg/mL), followed by *Salmonella typhi*, *Klebsiella pneumoniae*, *Micrococcus luteus* and the fungal strain *Candida albicans* (MIC50 = 19.77 mg/mL). Likewise, there was no antifungal activity observed against *Candida krusei*. A significant variation in the antimicrobial activities of the methanol and ethanol extracts was detected as the ethanol extract showed a higher inhibitory activity against the bacterial and fungal strains at similar concentrations than that detected by the methanol extract (Fig 2).

## Discussion

The pomegranate plant (*Punica granatum*) is one major fruit found in Jordan, consumed as palatable nutritious food and drink and used as a folk medicine for the treatment of many diseases [35–39]. The peels of this fruit have been reported to be rich in various bioactive components, in particular the phytochemicals, which in turn are responsible for their numerous reported health benefits including antioxidant and antimicrobial activities [18, 40, 41]. Despite the increased research interest in pomegranate peels, few studies have been made on pomegranate peels of Jordanian origin. Moreover, factors such as variations in the pomegranate cultivar, geographical region, and extraction methods affect the phytochemical composition and concentrations of pomegranate peels, and hence their health outcomes [11, 13]. Therefore, it is imperative to examine the phytochemicals; both qualitatively and quantitatively and the beneficial biological activities such as antioxidant capacities and antimicrobial activities of the Jordanian pomegranate peels extracted with various solvents.

The qualitative phytochemical screening showed the presence of several phytochemical classes (phenols, flavonoids, anthocyanins, coumarins, quinones, tannins, saponins, steroids, triterpenoid, and alkaloids) in various pomegranate peel extracts. Among the various pomegranate peel extracts used, the ethanol extract was the only extract that showed positive detection for all phytochemical classes tested. The results clearly indicate that ethanol was the most optimal solvent for extracting the various bioactive components from Jordanian pomegranate peels. Previous studies done on the Jordanian pomegranate peels showed similar results in which ethanol (absolute or diluted) was the best solvent for the extraction of their phytochemicals [18]. These results demonstrate that active compounds present in plant materials differ in their polarity, and their extraction depends greatly on the choice of solvent used and the method of extraction applied [19, 20].

Studies conducted on pomegranate peels have reported that among the various phytochemical classes present in pomegranate peels, phenols, flavonoids, and tannins were considered the major phytochemical classes found in pomegranate pulps [18, 40, 42]. Hence, the quantitative analyses of these phytochemical classes were determined. Different quantities of TPC, TFC, and tannins as HT and CT were detected in the various extracts of pomegranate peels. Again, the ethanol extract exhibited the highest TPC, TFC, HT, and CT contents compared to the rest of the extracts tested. This was in agreement with studies of Altarawneh et al. [18] who reported that the ethanol extract of the Jordanian pomegranate peels had the highest TPC and TFC compared to pomegranate peels’ water and acetone extracts and Kennas and Amellal-Chibane [43] who revealed that the HT content of the ethanol extract (682.39 ± 5.80 mg TAE/g DW) was high and similar to that detected in the aqueous methanolic extract (690.12 ± 13.53 mg TAE/g DW). The CT content was also detected in this study; however, its levels were lower than that of HT content and were in contrast to previous reports [43–45]. One possible explanation for this may be due to the variations in the extraction methods, in particular the polarity of the solvent employed for tannins extraction [42]. Nonetheless, both the qualitative and quantitative tests demonstrated the richness of the Jordanian pomegranate peels of phenolic compounds, in particular the phenols, flavonoids, and tannins.

Besides their richness in polyphenolic compounds, several studies reported the strong antioxidant capacities of pomegranate peels [46–49]. Indeed, the current study showed strong antioxidant activities (DPPH, ABTS^+^, and FRAP) exhibited by the various Jordanian pomegranate peel extracts. The highest scavenging effect against DPPH radicals was exhibited by the water extract, while the ethanol and methanol extracts demonstrated the highest scavenging effects against ABTS^+^ radicals. As for FRAP assay, the strongest reducing activity was achieved by the aqueous methanol extract. It is important to note that the antioxidant activities of any plant extract do not depend only on the composition of the extract, but also on the conditions of the test used and the various modes of action of the antioxidant activities [30, 50, 51]. For DPPH method, the presence of a hydrogen donor from an antioxidant results in the reduction of the stable DPPH radicals and the decrease in absorption intensity [14]. ABTS^+^ free radical assay, on the other hand, measures the antioxidant activity of both aqueous phase radicals and lipid peroxyl radicals. This assay does not depend only on the concentration of phenolics present in the extracts, but also on the structure and interaction between the different antioxidants [25]. While the FRAP assay measures the ability of the antioxidants in plants to reduce the Fe^+3^–2,4,6 tripyridyl-s-triazine (TPTZ) complex to the ferrous form (Fe^+2^) [29]. These various antioxidant activities demonstrated by various Jordanian pomegranate peel extracts in this study indicate how differently their antioxidants reacted with the different radicals tested, emphasizing the importance of testing the antioxidant activities with multiple assays. This was further supported by the Pearson’s correlation coefficient analysis.

Pearson’s correlation analysis showed a significant correlation between TPC and TFC (*P* < 0.01), indicating that flavonoids are the major polyphenolic compounds found in the Jordanian pomegranate peel extracts. This finding was in line with studies of More and Arya [52] and Kennas and Amellal [43]. Moreover, the strong and significant correlations between TPC and ABTS^+^ (*P* < 0.05) indicate that TPC are potent antioxidants against these radicals. The non-significant results between TPC and the other antioxidant assays tested (DPPH and FRAP) suggest that compounds other than the polyphenols found in pomegranate peel extracts may have attributed to the antioxidant capacities against these methods. Pearson’s correlation analysis also indicates that tannins (CT and HT) are not the major polyphenolic compounds found in pomegranate peels and hence are not responsible for the observed antioxidant activities. These unexpected correlations have been reported in other crude plant extracts [53]. The presence of a mixture of bioactive compounds in the crude extracts may have caused various types of interactions with the free radicals, hence the mixed outcomes [53]. Moreover, other bioactive families such as alkaloids, anthocyanins, and terpenoids, among others may be responsible for the antioxidant activities and hence should not be excluded.

Generally, extracts with high phenolic content and high antioxidant activities also exhibit high antimicrobial activities [46, 54, 55]. The ethanol and methanol extracts of the pomegranate peels showed the highest inhibition activity against the microbial strains *Pseudomonas aeruginosa*, *Klebsiella pneumoniae*, and *Micrococcus luteus* and the fungal strain *Candida albicans*. The phytochemical compounds retain a devastating effect against various pathogenic microorganisms and harbor potent healing properties for different diseases [34, 47, 48]. Dahham et al. [56] have reported a robust effect of the ethanol pomegranate peel extract against different microbial strains. Consistent with our results, Rosas ‐Burgos et al. [57] have demonstrated a high antimicrobial activity of ethanol extracts of pomegranate peels against both Gram-positive and Gram-negative bacterial strains as well as moderate antifungal activity against different fungal species.

## Conclusion

In conclusion, the current study revealed the richness of the Jordanian pomegranate peel extracts with various phytochemical classes, particularly the phenols, flavonoids, and tannins, with strong and promising antioxidant and antimicrobial potentials. The study also showed that ethanol was the most suitable solvent for pomegranate peel extraction with the highest antioxidant and antimicrobial activities. Pearson’s correlation coefficient further demonstrated the strong association between TPC, TFC, and ABTS^+^, but also indicated the need for further investigation for other bioactive components that may be responsible for the antioxidant activity of pomegranate peel extracts.

## Supporting information

S1 Data All data for the determination of antimicrobial activity are available in the supporting information file. (XLSX)
